# Whole genome sequencing revealed high proportions of ST152 MRSA among clinical *Staphylococcus aureus* isolates from ten hospitals in Ghana

**DOI:** 10.1128/msphere.00446-24

**Published:** 2024-11-20

**Authors:** Beverly Egyir, Christian Owusu-Nyantakyi, Alfred Bortey, Grebstad Rabbi Amuasi, Felicia Amoa Owusu, William Boateng, Hawawu Ahmed, Justice Kwesi Danso, Agnes Akosua Gyamaah Oclu, Quaneeta Mohktar, Georgina Tetteh-Ocloo, Harold Amegbletor, Kwabena Fosu, Francis Kwame Morgan Tetteh, Solomon Asante-Sefa, Oliver Nangkuu Deberu, Kennedy Mensah Osei, Joana Twasam, Sarkodie Kodom, Esther Gyinae, James Sampah, Nicholas Dzifa Dayie, Noah Obeng-Nkrumah, William Addo Mills-Pappoe, Gifty Boateng, Pernille Nilsson, Harriet Affran Bonful, Bright Adu, Rene S. Hendriksen

**Affiliations:** 1 Department of Bacteriology, Noguchi Memorial Institute for Medical Research, University of Ghana, Accra, Ghana; 2 Eastern Regional Hospital, Koforidua, Ghana; 3 St. Martin de Porress Hospital, Eikwe, Ghana; 4 Bolgatanga Regional Hospital, Bolgatanga, Ghana; 5 37 Military Hospital, Accra, Ghana; 6 Sekondi Public Health Laboratory, Effia Nkwanta Regional Hospital, Takoradi, Ghana; 7 Tamale Teaching Hospital, Tamale, Ghana; 8 Lekma Hospital, Accra, Ghana; 9 University of Ghana Hospital, Legon, Ghana; 10 Korle bu Teaching Hospital, Accra, Ghana; 11 St. Patrick’s Hospital, Ofinso, Ghana; 12 Department of Medical Microbiology, University of Ghana Medical School, Korle-Bu, Ghana; 13 Department of Medical Laboratory Sciences, School of Biomedical and Allied Health Sciences, University of Ghana, Accra, Ghana; 14 Clinical Laboratory Unit, Institutional Care Division-Ghana Health Service, Accra, Ghana; 15 Public Health Reference Lab, Korle-Bu, Ghana; 16 Research Group for Global Capacity Building, National Food Institute, WHO Collaborating Centre (WHO CC) for Antimicrobial Resistance in Foodborne Pathogens and Genomics, FAO Reference Laboratory (FAO RL) for Antimicrobial Resistance, European Union Reference Laboratory for Antimicrobial Resistance (EURL-AR), Technical University of Denmark, Kongens Lyngby, Denmark; 17 Department of Epidemiology and Disease Control, School of Public Health, University of Ghana, Accra, Ghana; 18 Department of Immunology, Noguchi Memorial Institute for Medical Research, University of Ghana, Accra, Ghana; University of Nebraska Medical Center College of Medicine, Omaha, Nebraska, USA

**Keywords:** whole genome sequencing, ST152 methicillin-resistant *S. aureus*, Africa

## Abstract

Previous studies in Ghana indicated low prevalence of methicillin-resistant *Staphylococcus aureus* (MRSA) and predominance of ST152 methicillin-susceptible *S. aureus* (MSSA) among clinical isolates. ST152 MRSA clones are associated with severe infections and epidemics. Using whole genome sequencing (WGS), 159 *S*. *aureus* isolated from clinical sources (wound, blood, urine, ear, abscess, umbilical cord, eye, vaginal samples, and others) from 10 hospitals across Ghana were investigated. *mecA* (gene for methicillin resistance) was detected in 38% of the isolates. Panton–Valentine leucocidin toxin (PVL) gene occurred in 65% isolates, with 84% of the MRSA’s harboring the PVL gene. ST152 was the major clone, with 74% harboring the *mecA* gene. Other MRSA clones detected were ST5, ST5204, ST852, and ST1. MSSA clones included ST3249, ST152, ST5, ST1, and ST8. Twenty-three genes encoding resistance to 12 antimicrobial classes were observed with *blaZ* (97%) being the most prevalent. Other predominant resistance genes included *tetK* (46%), *cat* (42%), and *dfrG* (36%) encoding resistance for tetracyclines, phenicols, and diaminopyrimidine, respectively. Virulence genes for enterotoxins, biofilms, toxic-shock-syndrome toxins, hemolysins*,* and leukotoxins were also detected. Phylogenetic analysis revealed a shift in the dominant clone from MSSA ST152 to MRSA ST152 over the past decade. The study provides valuable insights into the genomic content of *S. aureus* from clinical sources in Ghana. The finding of ST152 MRSA in high numbers suggests a shifting epidemiological landscape of these pathogens and continuous surveillance using robust tools like WGS is needed to monitor the rise and spread of these epidemic clones in the country.

**IMPORTANCE:**

Since its emergence in 1959, MRSA has been a significant public health concern, causing infections in both clinical and community settings. Patients with MRSA-related infections experience higher mortality rates due to its ability to evade antimicrobials and immune defenses. In Ghana, understanding the molecular epidemiology of MRSA has been hindered by the lack of appropriate laboratory infrastructure and the limited capacity for molecular data analysis. This study, the largest genomic study of *S. aureus* in Ghana, addresses this gap by utilizing whole genome sequencing to examine the diversity of circulating *S. aureus* strains from 10 hospitals. Our findings highlight the predominance of pandemic clones, particularly ST152, and the notable transition of ST152 MSSA to ST152 MRSA over the past decade. The findings from this study supports AMR surveillance efforts in Ghana and emphasize the importance of implementing genomic surveillance using WGS to comprehensively monitor the rise and spread of multi-drug-resitant organisms such as MRSA in the country.

## INTRODUCTION

*Staphylococcus aureus* (*S. aureus*) is a common pathogen that is associated with minor superficial wound infections to severe and potentially life-threatening conditions such as bacteremia, endocarditis, and necrotizing pneumonia (1). This pathogen has been identified by the World Health Organization as a high-priority subject for extensive research on its epidemiology and pathogenesis due to its remarkable combination of multidrug resistance and hypervirulent traits (2). In both hospital and community environments, infections caused by methicillin-resistant *S. aureus* (MRSA) often present treatment challenges, resulting in significantly higher rates of morbidity and mortality (3).

Several studies carried out have revealed the presence of diverse clones of *S. aureus* circulating worldwide with some strains being detected globally while others have a more localized distribution (4, 5). In Central and West Africa, *S. aureus* clones ST30, ST121, and ST152 have been consistently reported as the most prevalent clones (6–8). A significant proportion of these clones are known to carry Panton–Valentine Leukocidin (PVL) genes, with prevalence ranging from 9.1% to 100% in ST30 clones, 50%–93% in ST121, and 97%–100% in ST152 clones (6–8).

In Ghana, the ST152 clone has traditionally been identified as methicillin-sensitive *Staphylococcus aureus* (MSSA) in several previous studies with a reported prevalence range of 27% to 32% (9, 10). However, recent studies utilizing whole-genome sequencing (WGS) on few isolates have reported the emergence of ST152 MRSA among isolates from surgical site infections and farm attendants (6, 11). These MRSA strains were found to carry genes predominantly encoding resistance to penicillin, tetracyclines, and chloramphenicols. Additionally, they exhibited a diverse array of virulence genes, including leukotoxins, enterotoxins, and PVL toxin (6). As a result of its notable epidemiological and clinical implications, ST152 MRSA has gained considerable attention (12). The clone has been associated with outbreaks, severe infections, and demonstrates high virulence potential (13).

Whole Genome Sequencing (WGS) has revolutionized the study of bacterial epidemiology, offering high-resolution genomic information that can unravel genetic relationships between strains, identify resistance and virulence genes, and detect mobile genetic elements (14). Despite the value of WGS, reports on the utilization of this robust tool for AMR surveillance are limited on the African continent. In order to enhance our understanding of genomic epidemiology, clonal relatedness, and the profile of resistance and virulence genes, this study employed WGS to analyze a larger collection of clinical *S. aureus* isolates obtained from diverse sources from ten hospitals across Ghana.

## RESULTS

### Distribution of *S. aureus* isolates

**Fig 1:**
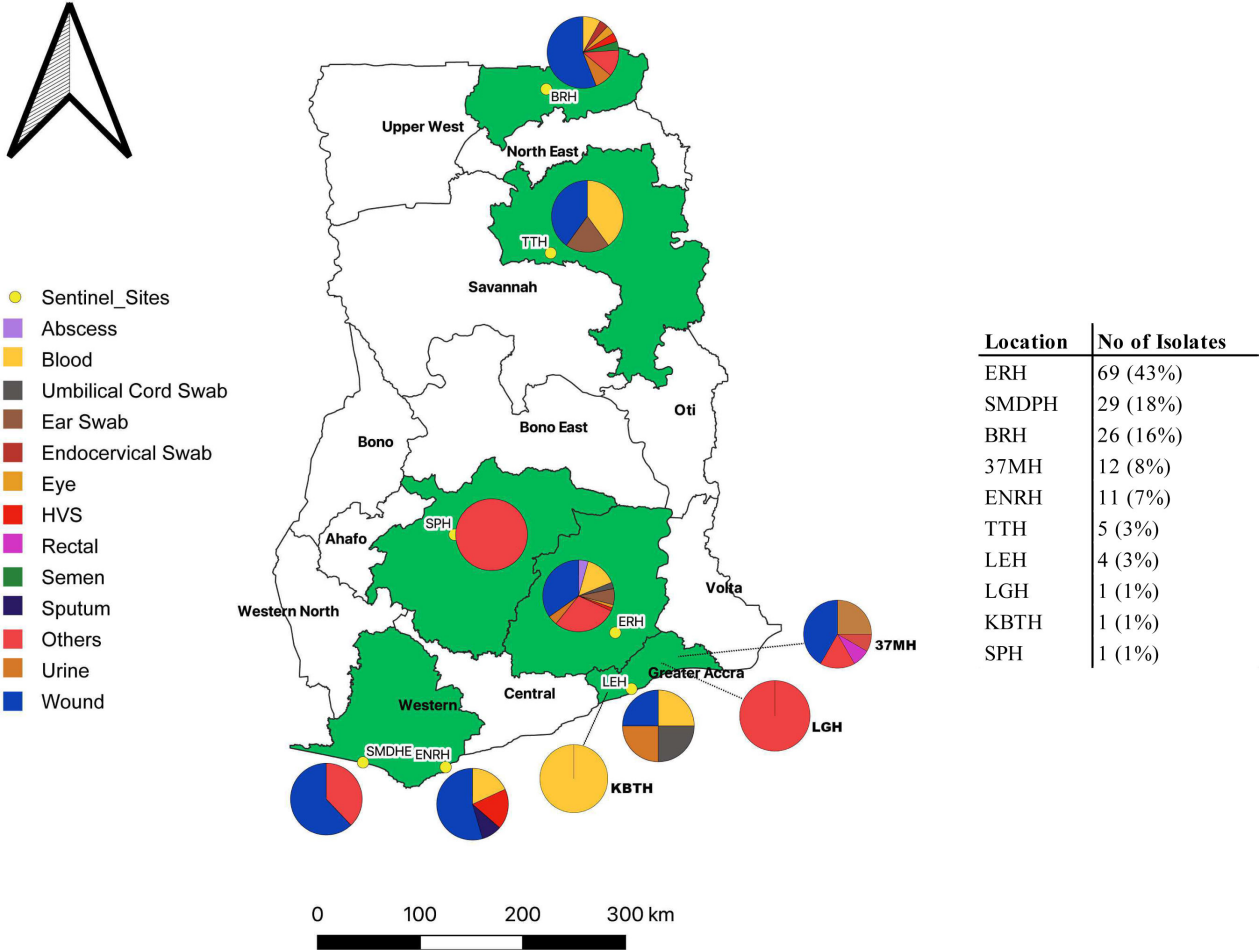
Geographical locations and clinical sources of the 159 *S. aureus* isolates. ERH, Eastern Regional Hospital; SMDHE, St. Martin De Porres Hospital Eikwe; BRH, Bolgatanga Regional Hospital; 37MH, 37 Military Hospital; ENRH, Effia Nkwanta Regional Hospital; TTH, Tamale Teaching Hospital; LEH, Lekma Hospital; LGH, Legon Hospital; KBTH, Korle-bu Teaching Hospital; SPH, St. Patrick’s Hospital.

Of the 159 *S*. *aureus* isolates investigated, the majority were obtained from Eastern Regional Hospital (43%, *n* = 69), St. Martin De Porres Hospital-Eikwe (18%, *n* = 29), and Bolgatanga Regional Hospital (16%, *n* = 26). Isolates originated mainly from wound (44%, *n* = 70), blood (11%, *n* = 18), and urine (6%, *n* = 9) samples. Figure 1 shows the geographical locations and clinical sources of the isolates.

### Array of virulence factors

The isolates harbored 50 different virulence genes that encode for 11 broad virulence factors (hemolysin, biofilm, siderophore, aureolysin, staphylokinase, leukotoxins, serine protease, immune evasion, enterotoxin, exfoliative toxin, and toxic shock syndrome toxin). The hemolysin encoding genes (*hlgA*, *hlgB, hlgC, hld, hla*), biofilm-forming genes (*icaA, icaB, icaC, icaD*) and aureolysin gene (*aur*) were the most predominant genes observed among the isolates. These genes were present among isolates from all sources. The biofilm-forming genes (*fnbA* and *clfA*) were found to be more prevalent among urine and blood isolates compared to those from wound, ear, and other sources. The PVL genes *lukF-PV* and *lukS-PV* were detected in 65% (*n* = 103) of the isolates, especially among isolates from the ear (85%) and wound (72%). Other toxin genes identified included those for enterotoxin, exfoliative toxin, and toxic shock syndrome-producing toxin. Table 1 summarizes the prevalence of all the virulence factors among the *S. aureus* isolates.

**TABLE 1:** Distribution of virulence genes of the *S. aureus* strains^*a*^^,^^*b*^

Virulence factors	Related genes	*N* = 159 (%)	Wound *n* = 71 (%)	Blood *n* = 18 (%)	Urine *n* = 9 (%)	Ear *n* = 6 (%)	Others *n* = 55 (%)
Hemolysin	*hlgA*	159 (100)	71 (100)	18 (100)	9 (100)	6(100)	55 (100)
	*hlgB*	159 (100)	71 (100)	18 (100)	9 (100)	6(100)	55 (100)
	*hlgC*	159 (100)	71 (100)	18 (100)	9 (100)	6(100)	55 (100)
	*hld*	158 (99)	70 (99)	18 (100)	9 (100)	6(100)	55 (100)
	*hla*	157 (99)	69 (97)	18 (100)	9 (100)	6(100)	55 (100)
	*hlb*	67 (42)	36 (51)	3 (17)	4 (44)	3 (50)	21 (38)
Biofilm	*icaA*	159 (100)	71 (100)	18 (100)	9 (100)	6(100)	55 (100)
	*icaB*	159 (100)	71 (100)	18 (100)	9 (100)	6(100)	55 (100)
	*icaD*	159 (100)	71 (100)	18 (100)	9 (100)	6(100)	55 (100)
	*icaC*	158 (99)	70 (99)	18 (100)	9 (100)	6(100)	55 (100)
	*fnbA*	144 (91)	67 (94)	18 (100)	8 (89)	5 (83)	46 (84)
	*fnbB*	131 (82)	61 (86)	16 (98)	7 (78)	5 (83)	42 (76)
	*clfA*	137 (86)	62 (87)	16 (98)	8 (89)	5 (83)	46 (84)
	*clfB*	135 (85)	65 (92)	14 (78)	8 (89)	5 (83)	43 (78)
	*cna*	52 (33)	29 (41)	5 (28)	4 (44)	1 (17)	13 (24)
Aureolysin	*aur*	158 (99)	71 (100)	18 (100)	9 (100)	6 (100)	54 (98)
Siderophore	*scn*	154 (97)	71 (100)	18 (100)	9 (100)	6 (100)	50 (91)
Staphylokinase	*sak*	129 (81)	60 (85)	14 (78)	8 (89)	5 (83)	42 (76)
Leukotoxins	*lukF-PV*	103 (65)	51 (72)	11 (61)	5 (56)	5 (83)	31 (56)
	*lukS-PV*	103 (65)	51 (72)	11 (61)	5 (56)	5 (83)	31 (56)
	*lukD*	92 (58)	35 (49)	14 (78)	5 (56)	3 (50)	35 (64)
	*lukE*	92 (58)	35 (49)	14 (78)	5 (56)	3 (50)	35 (64)
Serine protease	*splB*	93 (58)	35 (49)	14 (78)	5 (56)	3 (50)	36 (65)
	*splA*	89 (56)	33 (46)	13 (72)	5 (56)	3 (50)	35 (64)
	*splE*	54 (34)	22 (31)	8 (44)	4 (44)	2 (33)	18 (33)
Immune evasion	*edinB*	61 (38)	34 (48)	4 (22)	3 (33)	3 (50)	17 (31)
	*edinA*	8 (5)	2 (3)	2 (11)	-	1 (17)	3 (5)
	*edinC*	1 (1)	-	1 (6)	-	-	-
Enterotoxin	*sea*	54 (34)	22 (31)	7 (39)	3 (33)	2 (33)	20 (36)
	*seg*	29 (18)	10 (14)	4 (22)	1 (11)	1 (17)	13 (24)
	*sem*	29 (18)	10 (14)	4 (22)	1 (11)	1 (17)	13 (24)
	*sen*	29 (18)	10 (14)	4 (22)	1 (11)	1 (10)	13 (24)
	*seo*	29 (18)	10 (14)	4 (22)	1 (11)	1 (10)	13 (24)
	*sei*	29 (18)	10 (14)	4 (22)	1 (11)	1 (10)	13 (24)
	*seu*	29 (18)	10 (14)	4 (22)	1 (11)	1 (10)	13 (24)
	*seb*	16 (10)	5 (8)	4 (22)	1 (11)	1 (17)	3 (5)
	*sep*	15 (9)	6 (8)	3 (18)	-	-	7 (13)
	*seh*	14 (9)	5 (8)	3 (17)	2 (22)	2 (30)	4 (7)
	*seq*	14 (9)	3 (4)	3 (18)	3 (33)	1 (17)	4 (7)
	*sek*	14 (9)	3 (4)	3 (17)	3 (33)	1 (17)	4 (7)
	*ser*	8 (5)	4 (6)	2 (11)	-	-	2 (4)
	*sej*	8 (5)	4 (6)	2 (11)	-	-	2 (4)
	*sed*	6 (4)	2 (3)	2 (11)	-	-	2 (4)
	*sel*	3 (2)	2 (3)	1 (6)	-	-	-
	*sec*	3 (2)	2 (3)	1 (6)	-	-	-
	*sec3*	2 (1)	2 (3)	-	-	-	-
Toxic shock syndrome toxin	*tst*	4 (3)	-	-	-	-	4 (7)
Exfoliative toxin	*eta*	2 (1)	1 (1)	1 (6)	-	-	-
	*etb*	1 (1)	-	1 (6)	-	-	-

^*a*^ Others: abscess (3), vagina-HVS (6), cord (3), eye (2), rectum (1), sputum (1), semen (1), unknown source (38).

^*b*^ -, absence of genes among organisms.

### Resistome and mobile genetic elements of *S. aureus* isolates

A total of 23 different genes associated with 12 antimicrobial families (beta-lactams, phenicols, tetracyclines, macrolides, diaminopyrimidines, aminoglycosides, lincosamides, fluoroquinolones, fosfomycins, sulfonamides, streptomycins, and disinfectants) were observed among the 159 isolates. Thirty-eight percent (*n* = 61) of the isolates were MRSAs harboring the *mec*A gene. The beta-lactam gene *blaZ* was the most common (97%, *n* = 154) resistance gene observed. Phenicol resistance gene (*cat*) was detected among 42% (*n* = 66) of the isolates. *tetK* (46%, *n* = 73), *tetL* (1%, *n* = 2), and *tetM* (1%, *n* = 2) were the common tetracycline genes detected. Macrolide resistance encoded by *erm(C)* (10%, *n* = 16) and *fex(B)* (4%, *n* = 6) genes were also observed. The *dfrG* gene associated with resistance to diaminopyrimidine antibiotics was present among 36% (*n* = 57) isolates. The least prevalent resistant genes detected were sulfonamide (*sul1;* 1%, *n* = 1), streptomycins (*str;* 1%, *n* = 1)*,* and quinolones (*qnrD3;* 1%, *n* = 1). Table 2 shows the distribution of resistance genes among the isolates.

**TABLE 2:** Distribution of resistance genes and clinical sources of *S. aureus* isolates^*a*^^,*b*^

Antibiotic family	Resistant genes	*N* = 159 (%)	Wound *n* = 71 (%)	Blood *n* = 18 (%)	Urine *n* = 9 (%)	Ear *n* = 6 (%)	Others *n* = 55 (%)
Beta-lactams	*blaZ*	154 (97)	70 (99)	18 (100)	9 (100)	6 (100)	51 (93)
	*mecA*	61 (38)	34 (48)	4 (22)	4 (44)	1 (17)	18 (33)
Phenicol	*cat*	66 (42)	37 (52)	5 (28)	3 (33)	2 (33)	19 (35)
Tetracycline	*tet(K*)	73 (46)	35 (49)	7 (39)	6 (67)	4 (67)	21 (38)
	*tet(L*)	2 (1)	-	-	-	-	2 (2)
	*tet(M*)	2 (1)	-	-	-	-	2 (2)
Diamino-pyrimidine	*dfrG*	57 (36)	24 (34)	9 (50)	3 (33)	3 (50)	18 (33)
Macrolides	*erm(C*)	16 (10)	9 (13)	2 (11)	-	-	5 (9)
	*fexB*	6 (4)	3 (4)	1 (6)	-	-	2 (4)
	*erm(B*)	1 (1)	-	-	1 (11)	-	-
Aminoglycoside	*aph(3')-III*	9 (6)	4 (6)	-	1 (11)	-	4 (7)
	*ant(6)-Ia*	9 (6)	4 (6)	-	1 (11)	-	4 (7)
	*aac(6')-aph(2''*)	5 (3)	4 (6)	-	-	-	1 (2)
	*aph(2'')-Ia*	1 (1)	-	-	1 (11)	-	-
Lincosamide	*msr(A*)	7 (4)	2 (3)	1 (6)	-	1 (17)	3 (7)
	*erm(B*)	1 (1)	-	-	1 (11)	-	-
Fosfomycin	*fosD*	3 (2)	1 (2)	1 (6)	-	-	1 (2)
Disinfecting agent	*qacJ*	1 (1)	1 (2)	-	-	-	1 (2)
	*qacA*	1 (1)	-	-	-	-	1 (2)
Fluoroquinolone	*qnrD3*	1 (1)	1 (2)	-	-	-	-
Streptomycin	*str*	1 (1)	-	-	-	-	1 (2)
Sulfonamide	*sul1*	1 (1)	-	-	-	1 (17)	-

^*a*^ Others: abscess (3), vagina-HVS (6), cord (3), eye (2), rectum (1), sputum (1), semen (1), unknown source (38).

^*b*^ -, absence of genes among organisms.

SCC*mec* typing revealed five different SCC*mec* types. Of the 61 *S*. *aureus* isolates harboring *mec*A gene, 84% (*n* = 51) of the *mec*A were borne on SCC*mec*_type_IVa(2B) followed by SCC*mec*_type_Vc(5C2&5) (12%, *n* = 7), SCC*mec*_type_V(5C2&5) (3%, *n* = 2), and SCC*mec*_type _ XIII(9A) (2%, *n* = 1).

The majority of SCC*mec*_type_IVa(2B) isolates belonged to the ST152 sequence type (82%, *n* = 41) and predominantly harbored *mec*A*, blaZ, cat,* and *tet(K*) resistance genes. However, ST5 was the primary strain associated with SCC*mec*_type_Vc(5C2&5), carrying *mec*A*, blaZ,* and *tet(K*) as the predominant resistance genes. (Further details on SCC*mec* and association with STs and resistance genes are shown in Tables S1 and S5 of the supplemental material.)

Plasmids belonging to 17 distinct replicon types were detected among the 159 *S*. *aureus* isolates. Of these, *rep16* was the most predominant (75%, *n* = 119), followed by *rep5a* (69%, *n* = 109), *rep7a* (65%, *n* = 103), *rep20* (19%, *n* = 30), *rep7c* (18%, *n* = 28), *rep21* (11%, *n* = 17), *rep10* (11%, *n* = 17), and *rep19* (7%, *n* = 11). The least observed replicons (1%) were *rep15*, *rep22*, r*ep24a*, *repUS43*, *repUS5*, *rep10b*, *rep13*, *rep5c,* and *rep5e.* (Further details on plasmids are shown in Table S2 of the supplemental material.)

The *bla*Z beta-lactam gene was detected in *rep*16 (86%, *n* = 106) and *rep*5a (97%, *n* = 106) and to a lesser extent *rep*19 (64%, *n* = 7) and *rep*20 (63%, *n* = 19). For the *rep*7a plasmids, 60% (*n* = 62) and 65% (*n* = 67) harbored *cat* and *tet(K*) genes, respectively. The *str* gene was also borne on a *rep*7a plasmid. The *rep*19 plasmids carried several resistance genes, including *blaZ* (64%, *n* = 7), *dfrG* (55%, *n* = 6), *fexB* (27%, *n* = 3), *aac(6')-aph(2''*) (9%, *n* = 1), and *fosD* (18%, *n* = 2). Among the *rep*10 plasmids, 29% (*n* = 5) harbored *tetK* resistance genes and 94% (*n* = 16) carried *ermC* macrolide resistance gene. The plasmids *rep*15, *rep*24a, *rep*22*, rep*US43, *rep*5e, *rep*10b and *rep*5c harbored no resistant genes. (Further details on the resistance gene distribution among plasmids are shown in Table S3 of the supplemental material.)

### Clonal diversity and resistome of *S. aureus* isolates

MLST analysis identified 36 different MLST types. Sequence types ST152 (36%, *n* = 58), ST5 (11%, *n* = 17), and ST3249 (11%, *n* = 17) were the most predominant. Other STs, including ST1 (4%, *n* = 7), ST15 (3%, *n* = 5), ST8 (3%, *n* = 5), ST72 (3%, *n* = 4), ST121 (3%, *n* = 4), and ST789 (3%, *n* = 4), were also found. Sixteen of the 159 *S*. *aureus* isolates had novel sequence types [ ST7869 (*n* = 3), ST8344 (*n* = 3), ST7796 (*n* = 1), ST7797 (*n* = 1), ST7866 (*n* = 1), ST7867 (*n* = 1), ST7868 (*n* = 1), ST8345 (*n* = 1), ST8346 (*n* = 1), ST8747 (*n* = 1), ST8748 (*n* = 1), and ST8752 (*n* = 1) ] first reported in this study. However, two other isolates could not be typed as they were missing some of the loci, and their STs were labeled as unknown (Table S4).

Among the MRSAs (*n* = 61), ST152 was the major clone (72%, *n* = 44), followed by ST5 (5%, *n* = 3), ST1 (3%, *n* = 2), ST852 (3%, *n* = 2), and ST5204 (3%, *n* = 2). The least represented sequence types (*n* = 1) included ST2021, ST 7796 , ST789, ST8, ST8345, ST8747, and ST88.

Among the MSSA strains (*n* = 98), ST3249 was the predominant clone (17%, *n* = 17), followed by ST152 (15%, *n* = 15), ST5 (14%, *n* = 14), ST1 (5%, *n* = 5), ST15 (5%, *n* = 5), ST121 (4%, *n* = 4), ST72 (4%, *n* = 4), ST8 (4%, *n* = 4), ST7869 (3%, *n* = 3), ST789 (3%, *n* = 3), ST789 (3%, *n* = 3), ST8344 (3%, *n* = 3), ST2021 (3%, *n* = 3), and ST88 (*n* = 2). The least represented sequence types (*n* = 1) included ST101, ST12, ST3248, ST3250, ST3251, ST4921, ST567, ST6095, ST669, ST707, ST7797, ST7866, ST7867, ST7868, ST8346, and ST8748.

**Fig 2:**
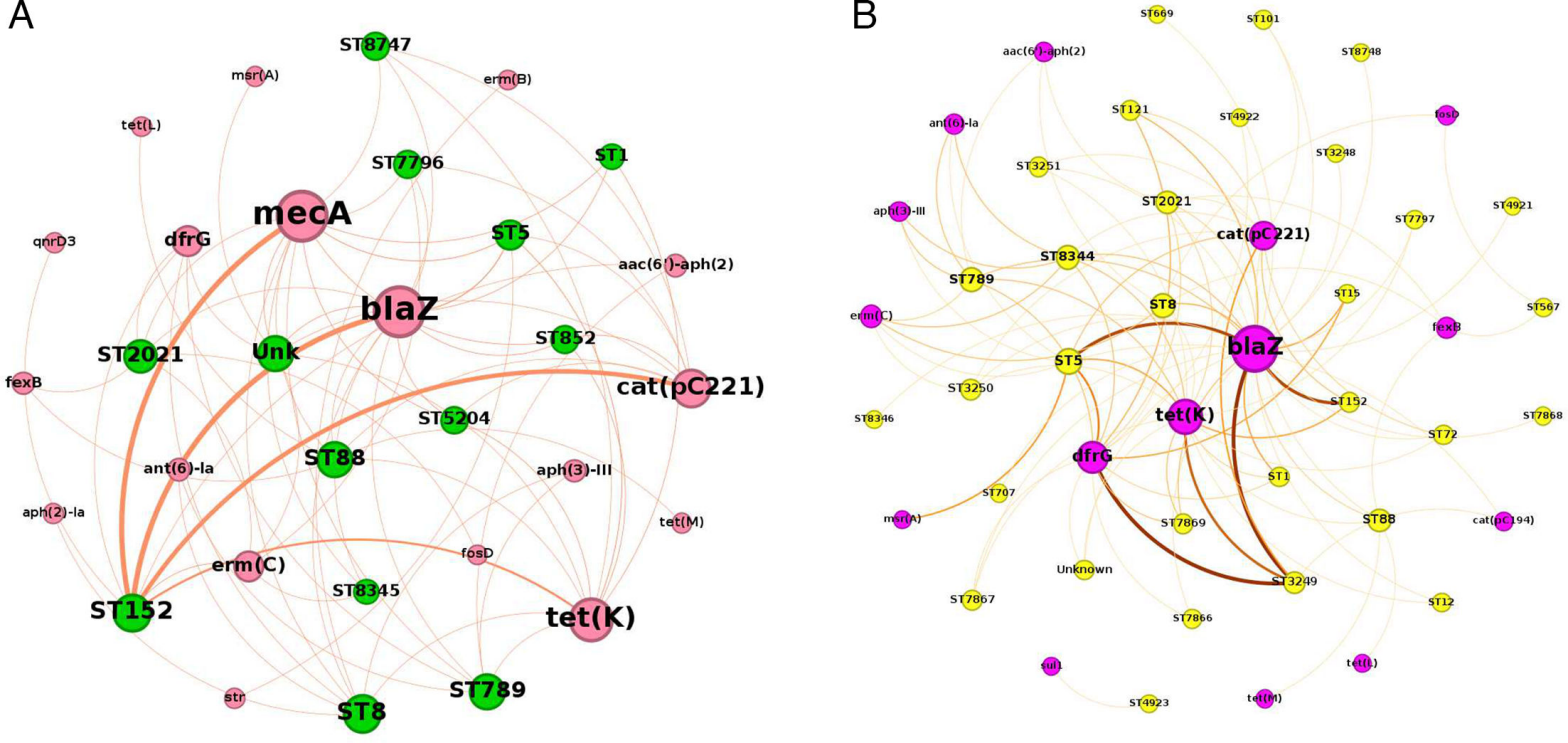
(**A**) Network analysis of the MRSA isolates showing correlation among the resistance genes and sequence types. The size of the nodes is proportional to the number of connections and the thickness of the edges express the strength of the correlations. Sequence types are represented by green nodes, while resistance genes are denoted by pink nodes. (**B**) Network analysis of the MSSA isolates showing correlation between the resistance genes and sequence types. The size of the nodes is proportional to the number of connections and the thickness of the edges express the strength of the correlations. Sequence types are represented by yellow nodes, while resistance genes are denoted by purple nodes.

The co-occurrence of resistance genes with sequence types within the MRSA cluster illustrated in Fig. 2A showed that the ST152 MRSA strains predominantly carried *mec*A*, blaZ, cat(pC221),* and *tet(K*) resistance genes, forming strong associations. Additionally, some ST152MRSAs carried genes such as *aac(6')-aph(2'')*, *dfrG*, *ermC*, and *qnrD3* represented as weaker connections. ST8 also demonstrated weaker connections to multiple resistance genes, encompassing *tetK, dfrG, ant(6)-Ia, blaZ, aph(3')-III, aph(2'')-Ia,* and *mec*A. Similarly, ST789 displayed several connections, linking to *aac(6')-aph(2''), ant(6)-Ia, dfrG, tetK, blaZ, aph(3)-III*, and *mec*A. Figure 2A provides further insights into the relationships within the MRSA cluster.

In contrast to the MRSA cluster, the strongest connections in the MSSA cluster (Fig. 2B) were observed among the ST3249 strains which mainly harbored *blaZ, tet(K), dfrG,* and *cat(pC221*) resistance genes. The ST5 strains primarily carried *blaZ, dfrG, msrA, tet(K*), and *cat(pC221*) showing strong connections. However weak associations were observed among ST5 and *ant(6)-Ia, aph(3')-III, ermC*, and *cat(pC194*) resistance genes. Similar to the ST8 MRSA, the ST8 MSSA isolates demonstrated multiple connections to *blaZ, aac(6')-aph(2'')], fexB, tetK, dfrG, fosD,* and *ermC*. The ST152 MSSA showed only three connections to resistance genes *blaZ, cat(pC221),* and *tet(K*). Figure 2B provides further insights into the relationships within the MSSA cluster.

Correlation analysis of resistance genes in the *S. aureus* isolates showed that the *blaZ* gene exhibited a strong positive correlation with the aminoglycoside gene *ant(6)-Ia* (r = 0.91) and a moderate correlation with *aph(3')-III* (r = 0.50), indicating their co-occurrence in the isolates. Similarly, the aminoglycoside resistance genes *aac(6')-aph(2"*) and *aph(3')-III* showed a high correlation (r = 0.73), indicating co-selection. Among the tetracycline resistance genes, *tet(L*) was positively correlated with both *erm(B)* (r = 0.57) and *tet(M)* (r = 0.57), while *fosB* displayed a moderate correlation with *fexB* (r = 0.50). (Further details on the co-occurrence or resistance genes are shown in Fig. S1 of the supplemental material.)

Our analysis also revealed distinct patterns within predominant sequence types (Fig. S2). Specifically, ST8 exhibited the highest resistance gene count, with a median of four, followed by ST152 and ST3249, each with a median of three. Conversely, minority strains, such as ST88, ST2021, ST789, and ST8344, demonstrated a higher burden of resistance genes, with a median of six. Concerning the virulence genes, ST5, ST72, and ST8748 stood out, carrying the highest numbers with medians of 29, 30, and 32, respectively. Additionally, analysis of the plasmid replicons revealed that ST8 harbored the most replicon types, with a median of four followed by ST8344 and ST3251, both with a median of five.

In comparing the ST152 isolates in the past decade, a noticeable trend emerged, indicating a general accumulation of genes particularly among the virulence genes of the *S. aureus* isolates over time (Fig. S3).

### Cluster analysis and comparative genomics

**Fig 3:**
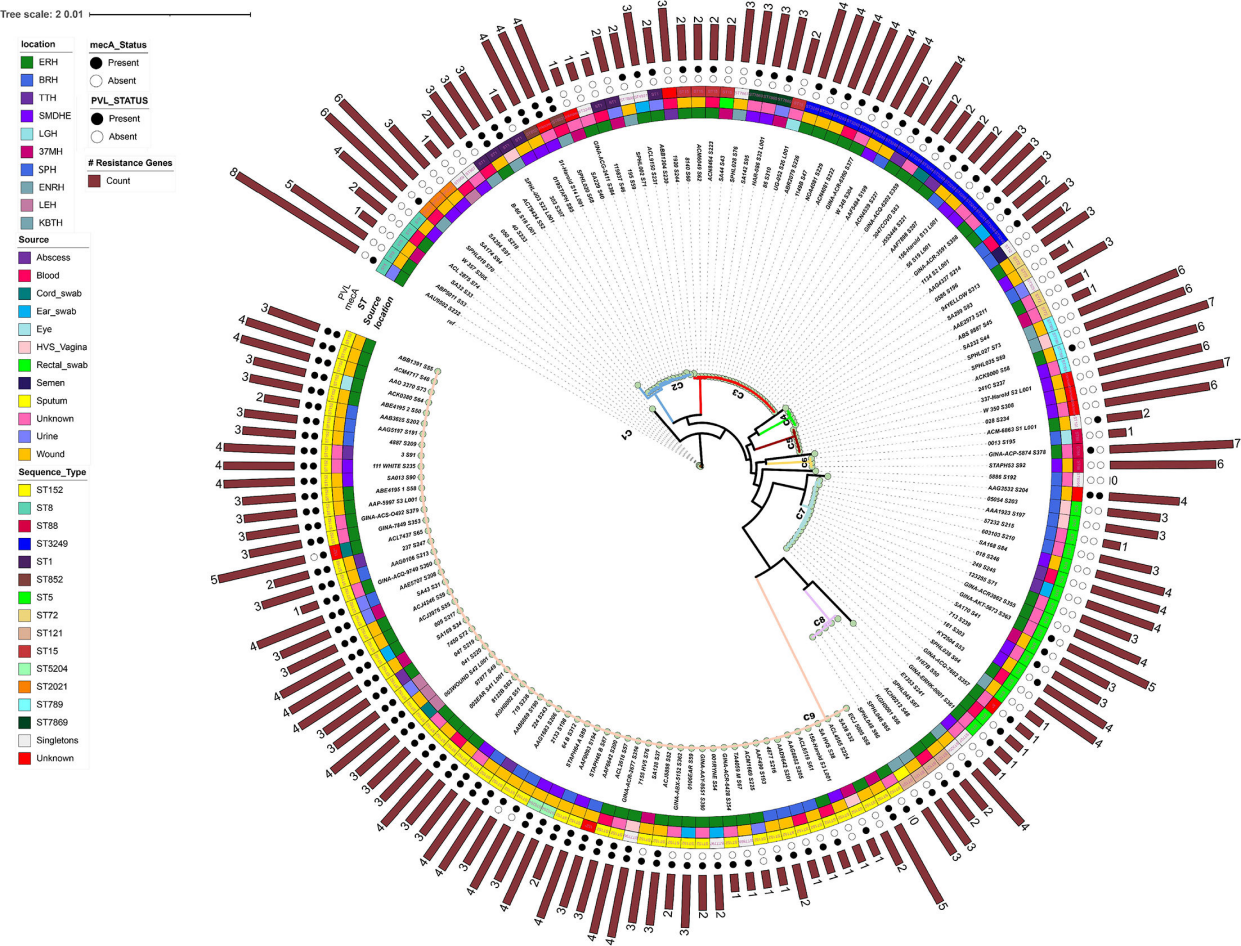
Core maximum likelihood phylogeny of the 159 *S. aureus* isolates. Phylogenetic tree was constructed based on single nucleotide polymorphisms aligned to reference strain NC_007795.1 The Phylogeny was inferred using Realphy. Nine major clusters were obtained (C1 to C9). **, isolates with novel ST. Rings annotated around the tree from the inner to outer; first ring (location), second ring (source), third ring (multi-locus sequence type), fourth ring (MRSA status), fifth ring (PVL status). The number of resistance genes harbored by each isolates is reported on the outermost ring as bar plot.

A high-resolution maximum likelihood phylogenetic tree was constructed using 24,140 core genome single nucleotide polymorphisms (SNP) (Fig. 3). The analysis revealed nine distinct clusters where isolates of similar sequence types (STs) were clustered together. In cluster 1 (C1), the majority of isolates exhibited an absence of both the PVL and *mec*A genes, with the exception of two isolates that were found to harbour the *mec*A gene. Within this clade, most of the isolates (except two) harbored several resistance genes, typically ranging from three to eight different genes. Cluster 2 (C2) consisted of isolates belonging to ST1, ST3248, and ST852. All isolates within Cluster 3 (C3) belonged to ST15, ST3249, ST7867, and ST7869. With the exception of two isolates, the majority in this cluster exhibited a positive presence of PVL and a negative status for *mec*A. Additionally, these isolates were observed to carry two to four other resistance genes. Strains in cluster 4 (C4) were negative for both PVL and *mec*A. These strains mainly belonged to ST72. Isolates within cluster 5 (C5) of ST789, while negative for both *mec*A and PVL, carried multiple other resistance genes, typically ranging between six and seven. The largest cluster, C9, predominantly consisted of strains of ST152, with the majority harboring both the PVL and *mec*A genes. The *mec*A positive ST152 strains harbored between three to five resistant genes while the *mec*A negative ST152 strains carried between one and two resistant genes. This predominant strain was detected across all sources and at all the sentinel sites.

**Fig 4:**
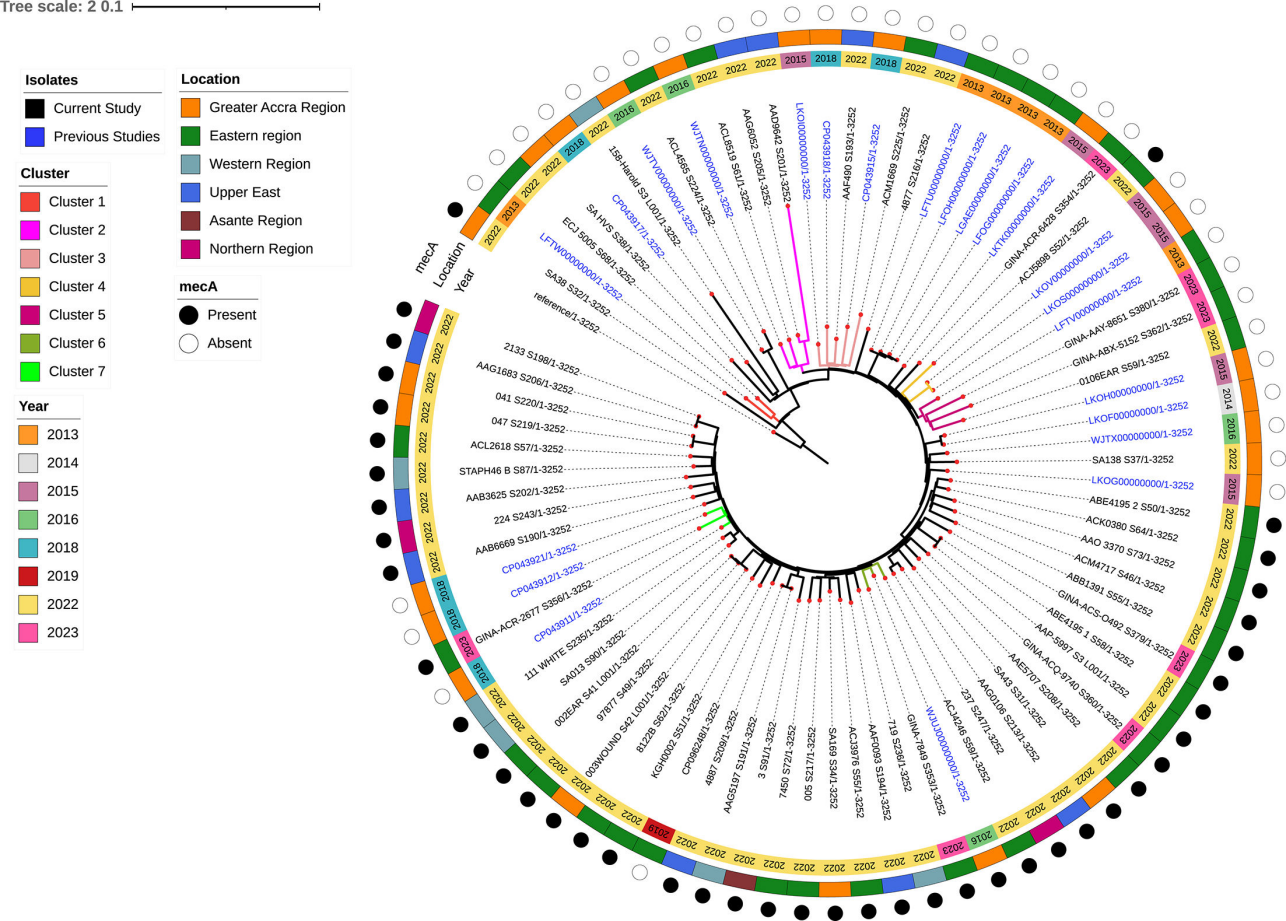
Single nucleotide polymorphisms-based phylogeny of the ST152 isolates from this study (*n* = 58) from clinical sources and ST152 isolates from previous Ghanaian studies (*n* = 24) recovered from clinical sources available at bv-brc.org. Tree was constructed using CSI-phylogeny aligned to reference strain NC_007795.1 and visualized using Interactive Tree of Life (iTOL). IDs highlighted in blue indicate strains from previous studies and those in black indicate strains from the current study. Inner ring shows the year of isolation. Middle ring shows regional location. Outer ring indicates MRSA status. Branches where current and previous strain cluster together are highlighted (Cluster 1 to Cluster 7).

To further reveal the evolutionary trajectory of the ST152 *S. aureus* isolates and understand how the strains from the current study compare to ST152 strains reported in prior studies, we constructed a maximum likelihood tree incorporating time and *mec*A status (Fig. 4). Cluster 1 comprised two isolates; one from previous studies isolated in 2013 and the other from this current study (2022). Cluster 2 comprised *mec*A negative isolates, including three from the current study (2022) and one from previous studies reported in 2016. In cluster 3, two isolates from this study (2022) clustered with three isolates from previous studies; one from 2015 and the remaining two from 2018. Cluster 4 contained one *mec*A positive strain from our study (2022) which clustered alongside two strains reported in 2015 all of which were *mec*A negative. Within cluster 5, a 2013 strain clustered with three strains from this study, specifically two from 2023 and one from 2022, all of which were *mec*A negative. Cluster 6 comprised three *mec*A positive strains, with one reported in 2016 and the other two originating from this study, one in 2022 and the other in 2023. In cluster 7, two *mec*A negative 2018 strains clustered with a *mec*A positive 2023 strain from this study.

Among the ST152 isolates, some of the strains from this current study were observed to share very close genetic relatedness with SNP differences below 25. Some strains from previous studies also shared SNP differences below 30 (Fig. S4).

## DISCUSSION

The evolution and spread of antimicrobial genes are of great concern to public health. Several studies in Africa and Ghana in particular have investigated the epidemiology of *S. aureus* using mainly antimicrobial susceptibility testing and Polymerase Chain Reaction (PCR) based methods. This study has generated high-resolution genomic data on *S. aureus* genetic diversity, antimicrobial resistance, and virulence genes. It notably augments genomic sequences available from Ghana and within the continent, thereby supplementing the currently sparse data set on this important pathogen.

We observed a predominance of *blaZ* (97%) known to be responsible for the beta-lactamase enzymes which hydrolyse the beta-lactam ring in penicillin antibiotics (15). This high proportion observed is in line with the previous studies in Ghana (6, 16) and elsewhere (17, 18). These rates observed are expected as penicillin has been used extensively to treat infections in humans and animals since the 1940s (19).

A concerning observation was the high proportion of *mec*A (MRSA) (38%) observed among the isolates with the majority originating from wound (56%, *n* = 34) and bloodstream (7%, *n* = 4) infections. Geographic variations in MRSA prevalence have been noted in previous reports, with rates in Europe ranging from 0.9% in the Netherlands to as high as 56% in Romania (20). It is important to highlight that in Asia, the Western Pacific, and Africa, the constrained AMR surveillance systems complicate the accurate assessment of true prevalence. Single studies in these areas have reported a wide range of rates between 0% and 73% (6, 21–23). The high prevalence of MRSA in this study is worrisome given the well-established association of these strains with treatment failure, increased healthcare costs, and elevated mortality rates (24). In Ghana, the primary antibiotic for treating *S. aureus* infections is flucloxacillin, a beta-lactam antibiotic (25). However, the rising prevalence of MRSA in infections raises concerns about the antibiotic’s efficacy. This concern is heightened by MRSA’s elevated production of beta-lactamases and its lower affinity for beta-lactams, emphasizing the potential challenges in effectively treating MRSA infections with flucloxacillin (26). A study conducted by Poilvache in 2020 highlighted these challenges, revealing that while flucloxacillin reduced bacterial counts and biomass in MSSA, it had no effects against MRSA (27). The situation is further complicated by the limited testing capacity in most laboratories in Ghana, hindering precise assessments of flucloxacillin’s effectiveness amidst emerging resistance trends (28, 29). Thus, enhanced antimicrobial surveillance and a reassessment of treatment strategies are crucial in addressing these evolving resistance patterns.

MRSAs are associated with the *SCCmec* cassette, a mobile genetic element harboring the *mec*A gene which confers beta-lactam resistance (30). In this study, *SCCmec*_type_IVa dominated among the MRSA strains (84%), aligning with other findings (31). We also observed that 82% of the *SCCmec*_type_IVa are associated with ST152 and predominantly carried the *mecA, blaZ*, *cat*, and *tet(K*) resistance genes. *SCCmec*_type_IVa is typically associated with community-acquired MRSA suggesting its widespread in our setting (32, 33). *SCCmec*_type_IVa MRSA strains have been associated with increased virulence and a higher incidence of severe infections such as bacteremia and ventilator-associated pneumonia (34).

The most prevalent tetracycline gene detected in this study is the *tetK* gene (46%). Tetracycline is one of the antibiotics prescribed for treating topical infections in Ghana which are often caused by *S. aureus* (25). The challenge is further compounded by the potential horizontal transfer of resistance genes to other bacteria. An interesting observation noted in this study was the high proportion of *tetK* (65%) genes carried by plasmid replicon type *rep7a* which has also been reported in other studies (35). The co-occurrence of tetracycline resistance genes *tet(L*) and *tet(M*) with the macrolide resistance gene *erm(B*) aligns with the findings of Congilosi et al. (36). This observation suggests the potential for increased transmission of resistance genes to other *S. aureus* strains, further limiting available treatment options (37).

Our study also identified a concerningly high prevalence (36%) of *S. aureus* isolates harboring the *dfrG* gene, for trimethoprim resistance. This finding is consistent with reports highlighting *dfrG* as the predominant trimethoprim resistance gene in *S. aureus* associated with human infections in Ghana and other African countries (6, 38, 39). In Ghana, co-trimoxazole, a combination of trimethoprim and sulfamethoxazole, has traditionally been a mainstay antibiotic for treating a wide range of infections including urinary tract infections and routine prophylaxis for people living with HIV (40, 41). This could explain the high prevalence of trimethoprim resistance in Ghana as observed in this study. Although a direct link between the observed *dfrG* prevalence and current co-trimoxazole prescription patterns in Ghana is yet to be established, the high resistance potential necessitates a re-evaluation of its continued use in treating infections. This is vital to ensure optimal patient outcomes and preserve the effectiveness of co-trimoxazole and commonly prescribed antibiotics in Ghana.

Hemolysin-encoding genes *hlgA*, *hlgB, hla* and *hld* were the most prevalent virulence genes (>99%). These genes are associated with the destruction of red blood cells of the host which is important for bacterial invasion and escape from the host immune response system (42). Similar to this study, research has shown that more than 95% of *S. aureus* strains harbor hemolysin encoding genes and their presence is independent of geographical location (43). These genes encode toxins that contribute to the pathogenicity of *S. aureus* and have been associated with severe clinical outcomes and mortality (44). The elevated prevalence of these hemolysin genes among clinical isolates signifies an increased potential for virulence and pathogenicity. Studies show that the more hemolysins are expressed, the higher the pathogenicity of the bacteria (45, 46). Although the presence of these genes can suggest a potential for increased virulence, their direct impact on clinical outcomes and patient management requires further investigation in conjunction with other clinical factors (45).

Vital toxins such as leukotoxins, enterotoxin, exfoliative toxins, and toxic shock syndrome toxin which constitute an important part of *S. aureus* virulence factors were observed among isolates in this study. The leukotoxin PVL, which is a bicomponent toxin encoded by the genes *lukF-PV* and *LukS-PV* was detected in 65% of the isolates originating from all sources except those from the rectum. Although PVL genes are frequently found in *S. aureus* isolates from Africa (6, 22, 47, 48), isolates recovered in Europe and Asia exhibit very low rates of PVL presence (49, 50). These variations in PVL toxin prevalence across continents may be attributed to host-related factors such as genetic variations in the C5a receptor, an immune signaling molecule that can influence immune cell susceptibility to PVL destruction (51). Mutations in this receptor can alter its binding affinity for PVL, which could explain lower PVL-negative *S.aureus* in some populations (51). Additionally, the presence of other unidentified virulence factors of *S. aureus* and environmental factors such as the humid nature of the African environment may influence bacterial survival and transmission, potentially contributing to the observed geographical distribution of PVL-positive *S. aureus* (52).

Noteworthy, the majority (83%) of the MRSAs in our study harbored PVL genes. PVL positive MRSAs have been associated with severe skin and soft tissue infections, necrotizing pneumonia, and other invasive infections with poor clinical outcomes (53, 54). In 2017, Yamuna et al. highlighted the potential risk of the emergence of multidrug-resistant MRSA with high virulence due to the presence of the PVL gene (55). The toxin has also been associated with several local epidemic outbreaks and serious illnesses such as recurrent furuncles, abscesses, or periorbital infection (1, 56, 57).

Of the 23 known staphylococcal enterotoxins, 18 were detected among isolates with *sea, seg, sem, sen, seo* and *sei* predominating. This is consistent with other studies reported in Ghana, Sudan, Brazil, and Iran (6, 58–60). These enterotoxins are usually implicated in food poisoning and can also cause intensive intestinal peristalsis (61). These findings necessitate the accurate identification of genes to monitor and regulate their dissemination and inform infection prevention strategies. Reports from studies that focused on the virulence factors of *S.aureus* have revealed that most *S.aureus* strains isolated from infections harbor one or more enterotoxin genes which are similar to the findings of this study (58, 59). Nonetheless, some studies have also shown that certain strains of the pathogen do not harbor any of these genes (62).

Among the global clones recovered in this study, ST152 was the most prevalent. ST152 has been reported as one of the most prevalent clones in Central and West Africa (6–8, 10). A large proportion of this clone is known to harbor PVL genes which is consistent with this study (9, 29). In addition, most of the ST152 clones reported in previous studies conducted in Ghana were MSSA (10, 63, 64). This is however in contrast to findings from this investigation as 74% of the ST152 clones are MRSAs.

The SNP-based analysis which compared ST152 strains from previous studies across various geographical locations to the ST152 strains in this study revealed that there is an observable shift in the dominant circulating clone from ST152 MSSA to ST152 MRSA. This suggests a possible broader, national-level dissemination of ST152 clones over time, potentially indicating a nationwide trend toward increased MRSA prevalence within this particular clone. The box plot and network analysis also showed this clone has been progressively acquiring additional virulence genes, along with plasmids over time. This observation raises significant concerns regarding its potential impact on public health as it suggests an increased capacity for causing more severe and antimicrobial-resistant infections. Also, the close genetic relatedness of some of the strains which shared less than 25 wgSNP differences indicates the potential for transmission within our settings (65). In a previous study (16), WGS revealed evidence of patient-to-patient transmission during an outbreak in a hospital setting in Ghana these occurrences underscore the significance of incorporating whole-genome investigations for AMR surveillance in conjunction with traditional methods. This integrated approach can facilitate the prompt identification of newly emerging clones with the potential to trigger outbreaks and to inform more efficient infection control strategies.

ST3249 MSSA was the second predominant clone observed in this study with strong association with genes encoding resistance to beta-lactams (*blaZ*)*,* tetracycline (*tetK*)*,* sulfonamides (*dfrG*), and chloramphenicol (*cat*) antibiotics. This clone was first reported in Ghana in 2017 (63) from the burns unit at a tertiary facility in the country (58). Other studies have reported its dominance among *S. aureus* isolates (6, 39). This clone now seems to be taking a seat as a predominant clone in Ghana (39, 63). Other sequence types that were also predominant in the study included ST5, ST8, ST1, ST121, and ST15, similar to what was observed in previous studies in Ghana (6, 16, 63).

Overall, this study contributes significantly to our understanding of the genomic characteristics of *S. aureus* in Ghana. The isolates exhibited a wide array of resistance and virulence genes. Additionally, high prevalence of toxin-producing genes, including those encoding Panton valentine leucocidin, hemolysins and enterotoxins was observed. The frequent circulating ST152 epidemic clone was predominantly methicillin-resistant. The phylogenetic analysis highlighted the potential transmission of *S. aureus* strains within our settings. These findings underscore the necessity for ongoing monitoring via an integrated genomic surveillance to detect emerging clones, respond swiftly to potential outbreaks as well as combat AMR.

## MATERIALS AND METHODS

### Study design

This cross-sectional study employed total enumerative sampling to investigate *S. aureus* isolates from ten sentinel hospitals in Ghana. Between February 2022 and October 2023, all cultures with suspected *S. aureus* from these hospitals during routine clinical procedures were submitted to the Noguchi Memorial Institute for Medical Research (NMIMR) for confirmation. These cultures originated from diverse clinical samples such as wound swabs, urine, blood, sputum, high vaginal swabs, abscesses, umbilical cord swabs, eye swabs, and ear swabs. Confirmation of the *S. aureus* isolates was performed at NMIMR using Matrix-assisted Laser Desorption/Ionization Time of Flight (MALDI-TOF) and then WGS was performed to obtain draft genomes. Epidemiological data associated with the isolates were obtained as documented in the hospital laboratory records.

### Isolation and identification of *S. aureus*

Upon arrival in NMIMR, isolates were inoculated on mannitol salt agar (Oxoid, UK) plate and incubated aerobically at 37°C for 16 to 24 hours. The overnight cultures were sub-cultured onto blood agar plates. Bacterial colonies presenting with distinct morphotypes of *S. aureus* were confirmed using the Matrix-assisted Laser Desorption/Ionization Time of Flight (MALDI-TOF) mass spectrometry (Bruker Daltonics, Germany) and Biotyper system 2.0 software at the genus [log(score) 1.7–2.0)] and species [log(score) ≥2.0] level.

### DNA extraction and whole genome sequencing

DNA of the confirmed *S. aureus* isolates was extracted using the QIAamp DNA mini kit (QIAGEN Inc. GmbH, Holden, Germany) following the manufacturer’s instructions. The concentration of the extracted DNA was determined using Qubit 4.0 Fluorometer assay kit (Thermo Fisher Scientific, MA) and diluted accordingly to obtain concentrations ranging between 10 and 60 ng/µL in a final volume of 30 µL. Libraries of the DNA were prepared using the Illumina DNA library prep - (M) Tagmentation kit (Illumina Inc., San Diego, CA). The quality and concentration of the libraries were assessed using the 2100 bioanalyzer systems (Agilent) and qPCR kappa library quantification kit (Roche, California) respectively. All libraries were normalized to a concentration of 2 ng/µL and pooled together. Pooled libraries were then loaded on a 2 × 150 cycle cartridge for sequencing on the Illumina Nextseq 2000 system (Illumina Inc., San Diego, CA). Sequenced files were retrieved after 72 hours.

### In-silico analysis of sequenced isolates using bioinformatic tools

To check the quality of sequences produced by the Nextseq 2000, raw read files were quality filtered using FASTQC v0.12.1 (66). Trimmomatic v.0.39 was used to trim adaptors and bases with quality score below 20 (67). Trimmed reads were de-novo assembled into contigs using Unicycler v.0.5.0 (68). The assembled genome was quality-checked with Quast software v.5.2.0 (69). Genomes with coverage below 20X and those with contigs greater than 300 were re-sequenced. All genomes used for post-sequencing analysis had coverages greater than 20X and contigs with more than 300 bases. Assembled sequence data were analyzed using Kmerfinder v.4.1 (70) to confirm bacteria species, CARD v.3.0.9 (71) and Resfinder v.4.1 (72) to identify resistance genes, Virulencefinder v.2.0 (73) and VFDB (74) to detect virulence genes and MLST v.2.0 (75) to determine their sequence types. Genomes with novel MLST sequences were submitted to PubMLST for new ST to be assigned. SCC*mec* finder was used to type SCC*mec* (76). Plasmidfinder v.2.1 (77) was used to detect plasmid sequences. Mob-recon from Mob-suite v3.1.8 (78) was used to extract plasmids from the assemblies. Subsequently, the extracted plasmids were analyzed for the presence of resistance genes using Resfinder. All the tools were used at default settings. Phylogenetic analysis based on differences in single nucleotide polymorphism (SNP) was conducted using CSI Phylogeny and Realphy with default settings (79, 80). Bactinspector v0.1.3 (https://gitlab.com/antunderwood/bactinspector/) was used to select the best reference for the genomes. The calculated trees were exported in newick format and then visualized and annotated using interactive Tree of Life (iTOL) (81). Cluster classification was based on Average Nucleotide Identity threshold of 99.5% using the Average Nucleotide Identity calculator (https://www.ezbiocloud.net/tools/ani). Other Ghanaian *S. aureus* genomes were downloaded from the Bacterial and Viral Bioinformatics Resource Center (https://www.bv-brc.org/) and compared with genomes from this study to assess evolutionary relationships based on SNP. Analysis tables were created using Excel v.16.82 while the box and whisker plots were generated using ggplot2 statistical package in R studio v.4.1. The co-occurrence network analyses, which represented the possible correlations between the resistance genes and sequence types, were created, and visualized in Gephi 0.10.1.

## Data Availability

The sequence data generated have been submitted and deposited in the NCBI database under the BioProject accession number PRJNA950924. The sequence data from bv-brc.org that were utilized in the analysis are accessible through specific accession numbers provided in the supplemental material (ST152 from previous studies).

## SUPPLEMENTAL MATERIAL

Fig. S1 msphere.00446-24-s0001.eps Correlation matrix of the resistance genes in the *S. aureus* isolates.

Fig. S2 msphere.00446-24-s0002.eps Total number of resistance genes, virulence genes, and plasmid replicon types distributed across the sequence types in the *S. aureus* isolates.

Fig. S3 msphere.00446-24-s0003.eps Total number of resistance genes, virulence genes, and plasmid replicon types in ST152 *S. aureus* isolates: current study (2022-2023) and previous study (2013-2019) in Ghana.

Fig. S4 msphere.00446-24-s0004.eps Pairwise distance matrix based on core SNPs among the ST152 strains isolated from this study and previously reported by other Ghanaians studies.

Legends msphere.00446-24-s0005.docx Contains legends for supplemental figures and tables.

Table S1 msphere.00446-24-s0006.xlsx Information on different types of Sccmec and their distribution among the *S. aureus* genomes.

Table S2 msphere.00446-24-s0007.xlsx Distribution of plasmid replicons among the *S. aureus* isolates.

Table S3 msphere.00446-24-s0008.xlsx Distribution of resistance genes harbored by plasmid replicons.

Table S4 msphere.00446-24-s0009.xlsx Distribution of sequence types of the *S. aureus* genomes.

Table S5 msphere.00446-24-s0010.xlsx Distribution of the Sccmec type, sequence types, and associated resistance genes.

ASM does not own the copyrights to Supplemental Material that may be linked to, or accessed through, an article. The authors have granted ASM a non-exclusive, world-wide license to publish the Supplemental Material files. Please contact the corresponding author directly for reuse.
