# Supplementary material for: Whole genome sequencing revealed high proportions of ST152 MRSA among clinical Staphylococcus aureus isolates from ten hospitals in Ghana
Source: mSphere. 2024 Nov 20;9(12):e00446-24. doi: 10.1128/msphere.00446-24 (PMC11656792; doi:10.1128/msphere.00446-24)
Supplement: Legends — Contains legends for supplemental figures and tables. [file msphere.00446-24-s0005.docx]

**Supplemental Figure Legends**

**FIG S1 :Correlation Matrix of the Resistance Genes in the *Staphylococcus aureus* Isolates**

The heatmap displays the correlation coefficients between various resistance genes, highlighting significant associations. Darker shades represent stronger correlations, while lighter shades represent weaker or no correlation.

**FIG S2:Total number of resistance genes (A), virulence genes (B) and plasmid replicon types (C) distributed across the sequence types in the *S. aureus* isolates.** Black dots represent outlier data points, black dashes represent the median, boxes represent the 25th, 50th an 75th percentile, and the whiskers inidicate the range of the data points

**FIG S3:Total number of resistance genes (A), virulence genes (B) and plasmid replicon types (C) in ST152 *S. aureus* isolates: Current study (2022 – 2023) and Previous study (2013 – 2019) in Ghana.** Black dots represent individual data points, black dashes represent the median, boxes represent the 25th, 50th an 75th percentile, and the whiskers inidicate the range of the data points

**FIG S4: Pairwise distance matrix based on core SNPs among the ST152 strains isolated from this study (labelled in black) and ST152 strains previously reported by other Ghanaians studies (labelled in red).** Colours in the heat map represent the number of SNP differences as shown in the key on the right

**Supplemental Table legends**

**Table S1: Distribution of SCCmec Types Among the *S.aureus* Isolates.**

The table shows the presence of various SCCmec types haoured by the mecA positive isolates. Entries marked with a dash ("-") indicate the absence of the respective SCCmec type in that isolate.

**Table S2: Distribution of Plasmid Replicons among the S.aureus Isolates**

The table shows the presence of various plasmid replicons across *S. aureus* isolates, identified by sequence type (ST). Each isolate is annotated with detected replicons, with absence indicated by a dash ("-").

**Table S3: Distribution of Resistance Genes Harboured by Plasmid Replicons**

The table shows the proportion of resistance genes harboured by the different plasmids present in the *S. aureus* genomes

**Table S4: Distribution of Sequence types of the *S. aureus* genomes**

The table shows the frequency and percentage of occurrence for each sequence type (ST) identified among the *S. aureus* genomes.

**Table S5: Distribution of the Sccmec type, Sequence types and Associated Resistance Genes.** The table presents the relationship between *Sccmec* types, the sequence types (STs) they are associated with, and the corresponding resistance genes detected.
